# Separation of mercuric ions using 2-thienylbenzimidazole/cucurbit[7]uril/iron-oxide nanoparticles by pH control

**DOI:** 10.1038/s41598-023-38199-2

**Published:** 2023-07-12

**Authors:** Falguni Chandra, Paltan Laha, Farah Benyettou, Tina Skorjanc, Na’il Saleh

**Affiliations:** 1 Department of Chemistry, College of Science, United Arab Emirates University, P.O. Box 15551, Al Ain, United Arab Emirates; 2 Zayed Bin Sultan Center for Health Sciences, United Arab Emirates University, PO. Box 15551, Al Ain, United Arab Emirates; 3 New York University Abu Dhabi, P.O. Box 129188, Abu Dhabi, United Arab Emirates

**Keywords:** Nanoparticles, Single-molecule fluorescence, Dynamic combinatorial chemistry

## Abstract

2-Thienylbenzimidazole (**TBI**)/cucurbit[7]uril (CB7) host–guest complex was used as a motif to significantly improve the turnover of γ-Fe_3_O_4_ magnetic nanoparticles for potential application in the separation of toxic mercuric ions in polluted water samples. The mechanism of restoring the original solid materials is based on applying the pH-controlled preferential binding of the CB7 host to the **TBI** guest. The analytical application of this concept has not been realized in the literature. The pH-controlled stimuli-responsive abilities were confirmed in aqueous solution by the three-order of magnitudes higher stability constant of the protonated **TBIH**^**+**^/CB7 complex (e.g., *K* = 4.8 × 10^8^ M^−1^) when compared to neutral **TBI**/CB7 complex (e.g., *K* = 2.4 × 10^5^ M^−1^), also manifested in an increase in p*K*_a_ values by ~ 3.3 units in the ground state. The supramolecular interaction and adsorption on iron oxide nanoparticles (NPs) were also spectroscopically confirmed in the solid state. The excited-state lifetime values of **TBI**/CB7NPs increased upon lowering the pH values (e.g., from 0.6 to 1.3 ns) with a concomitant blue shift of ~ 25 nm because of polarity effects. The time-resolved photoluminescent behaviors of the final solids in the presence of CB7 ensured pH-driven reusable systems for capturing toxic mercuric ions. The study offers a unique approach for the controllable separation of mercury ions using an external magnet and in response to pH through preferential binding of the host to guest molecules on the top of magnetic surfaces.

## Introduction

Recently, the most significant development of nanostructured materials with stimuli-responsive and switchable functions was achieved using supramolecular host–guest chemistry^1–3^. To bypass the synthetic hazards, the researchers focus on non-covalent interactions over the traditional organic covalent bonds^4–10^. The non-covalent interaction (e.g., dipole–dipole interactions, van der Waals interactions, and hydrogen bonding) played a leading role within the host–guest supramolecular approach where the small organic molecules (guest) held inside the nanocavity containing macrocycle host^4–10^. Inspired by the specificity of non-covalent interactions, we have researched developing host–guest-based advanced materials for water quality monitoring^11–13^.

**Figure 1:**
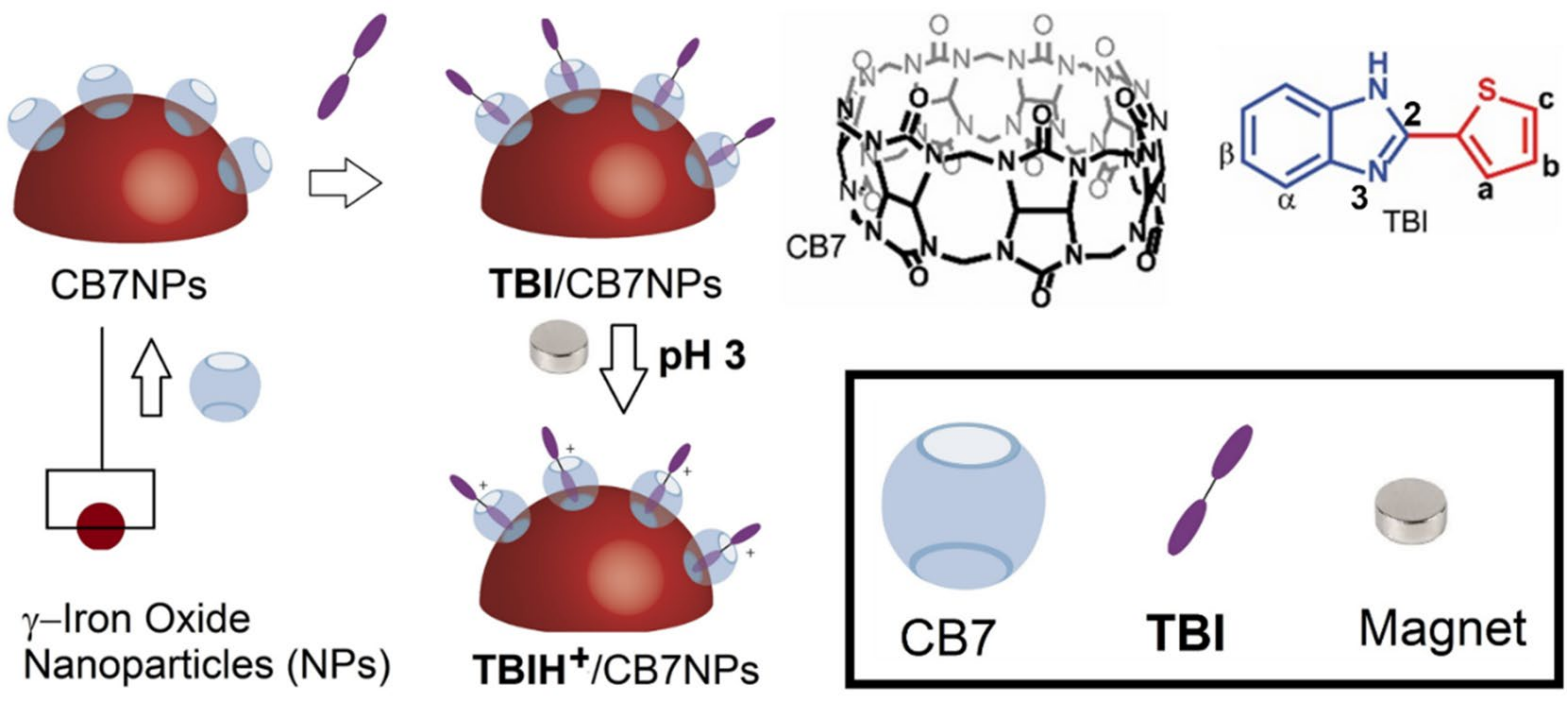
Schematic representation of pH-controlled **TBI**/CB7-loaded NPs. Chemical structures of the fluorescence probe **TBI** and the cucurbituril macrocycles, CB7, are also shown. Depending on the protonation/deprotonation of the nitrogen at position-3, two forms can exist of the coordinating ligand: **TBI** and **TBIH**^**+**^.

The application of cucurbiturils (CBn, *n* = 6, 7, 8, 10; Fig. 1) to interact with small fluorescent molecules was realized in literature where cucurbiturils modulate the p*K*_a_s (protonation states) of embedded guests such as benzimidazoles^14^. Consequently, the CB*n*-induced p*K*_a_ shifts have been shown to establish pH-driven control over the sequestration and release of guest molecules^14, 15^. Guest retention and release in response to pH stimuli have also been utilized to construct stimuli-responsive nanostructured materials^16^.

From another perspective, the dyes encapsulated by CB7 have been adsorbed on the surface of metal nanoparticles^17–21^ for drug delivery^18^ magnetic resonance imaging^20^, and the fabrication of solar cell materials^19^. The iron-oxide magnetic nanoparticles (γ-Fe_3_O_4_) have been specifically utilized for chemical detection and sensing^22^. It was used for mercury sensing using water-soluble cyclodextrin and solvents as the trigger for switching^11^.

In this work, we demonstrated the pH-responsive iron oxide nanoparticles (NPs) surface coated with nanocontainer CB7 which encapsulates a benzimidazole-based fluorescent dye (2-thienylbenzimidazol) **TBI**, Fig. 1^23^. Thus, the present supramolecular-based nanomaterials approach has much more potential to repeatedly use those solid materials by responding to pH triggers (as opposed to using a solvent)^11^.

The intensity measurements by fluorescence are not practical for the detection or separation application of undesired residues in water samples because the intensity is dependent on the concentration of the fluorophore. Thus, the time-resolved photoluminescence (PL) experiments were performed in the solid state of the **TBI**/CB7 host–guest supramolecular system on γ-Fe_3_O_4_ nanoparticles.

## Results and discussion

### Interactions of **TBI** with CB7 in solution

**Figure 2:**
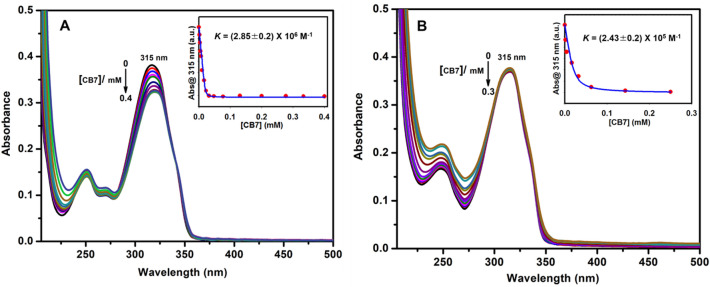
UV–visible absorption titration of **TBI** (20 μM with CB7) at different pH: (**A**) and (**B**) illustrate the binding with CB7 at pH 2 and 7, respectively. The inset shows the corresponding titration curve and the 1:1 binding fit (solid line) with the corresponding binding constant, *K,* for the data in (**A**) and (**B**). For clarity, the spectra at different CB7 concentrations are shown in different colors. The numbers are the corresponding maxima (in nanometers).

The encapsulation of **TBI** by CB7 is evidenced from UV–visible absorption measurements in water at pH 2 and 7 in Fig. 2 (see also pH-titration data below). CB7 encapsulates the protonated or neutral **TBI** molecules, causing a redshift in their absorption spectra from 315 to 320 nm.

**Figure 3:**
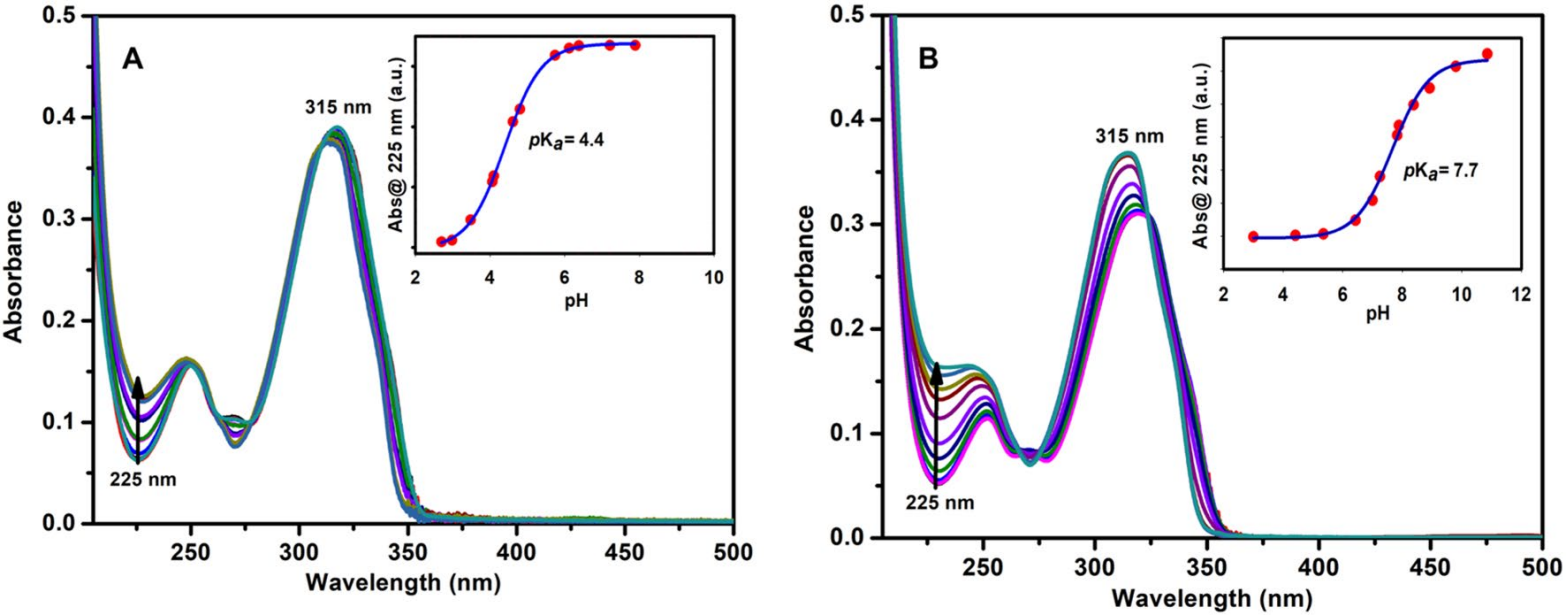
Ground state pH-titration of (**A**) 20 µM **TBI** only and (**B**) 20 µM **TBI** with 3 mM CB7. Inset showing corresponding p*K*_a_ values. For clarity, the spectra at different pH are shown in different colors. The numbers are the corresponding maxima (in nanometers).

Proton-NMR spectroscopy also confirmed the complexation of TBI protonated forms to CB7 at pD 2 (Fig. S1 in the Supporting Information). Previous reports on other benzimidazole derivatives demonstrated similar host-induced shifts on account for the shielding of the protons of the benzimidazole guest molecules when encapsulated in the electron-donating cavities of CB7 (e.g., fuberidazole, where the sulfur atom is replaced by oxygen)^14, 24–27^. Two pH values, pH 2 and 7, were selected for the binding experiments (Fig. 2A,B) based on the pH titration data in the absence (Fig. 3A) and the presence of CB7 (Fig. 3B). The complex affinities were also estimated at each pH (e.g., *K* = 2.8 × 10^6^ M^−1^ at pH 2 and *K* = 2.4 × 10^5^ M^−1^ at pH 7). pH and binding titration experiments were already conducted by monitoring the fluorescence emission of **TBI** upon varying the pH or the concentrations of CB7^25^.

Table S1 in the Supporting Information summarizes the different binding constants measured using NMR, UV–visible absorption, and fluorescence techniques. Compared to that binding constant measured by UV–visible, the value measured by NMR was lower by two orders of magnitude, which is expected because of the concentration differences (millimolar in NMR data versus micromolar in optical data). Binding constants of the excited states (PL method) to CB7 were also much lower by one-to-two orders of magnitudes (depending on the pH values) than those for the ground state’s binding affinities (UV method), which is unsurprising^14^. The pH-titration plot for the free **TBI** in Fig. 3A gave p*K*_a_ values in the ground state because of the nitrogen protonation at position 3, which increased by ~ 3.3 units upon the addition of CB7 (Fig. 3B).

**Figure 4:**
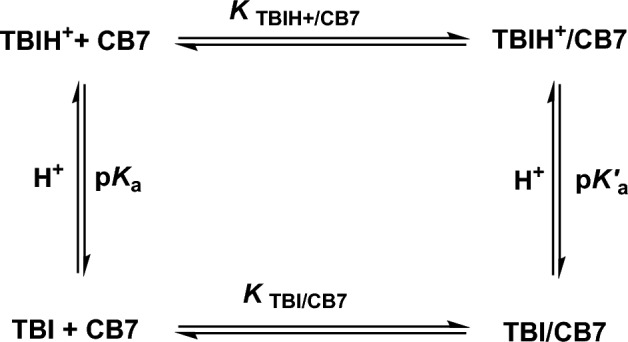
Four-state complexation model of the neutral and protonated **TBI** with CB7.

Because the protonation of CB7 occurs at pH 2, which affects the binding between the protonated **TBI** and CB7 by analogy to similar benzimidazoles^14^, a more accurate binding constant at pH 2 should be calculated from the corresponding p*K*_a_ shift using the previously reported thermodynamic relation^14^, giving a value of *K* = 4.8 × 10^8^ M^−1^. The relation is based on a four-state model, which involves (a) the uncomplexed and unprotonated **TBI** (guest), (b) the uncomplexed protonated **TBIH**^**+**^ (guest), (c) the unprotonated **TBI**/CB7 (host–guest) complex, and (d) the protonated **TBIH**^**+**^/ CB7 (host–guest) complex, which are all connected through a thermodynamic cycle as illustrated in Fig. 4.

The CB7-induced p*K*_a_ shifts are unsurprising and agree with previous reports on other benzimidazole derivatives^14, 24–27^. To ensure a controlled release and retention of the guest from the host cavity, the binding affinity of the protonated and neutral forms of **TBI** to CB7 must be different. The present study confirms the three-order magnitudes difference. Consequently, it was decided to opt for this supramolecular approach to develop nanoparticles whose structures could be switched repeatedly in response to pH values while being monitored utilizing time-resolve PL measurements (see below).

### Interactions of **TBI** with CB7NPs in the solid state

**Figure 5:**
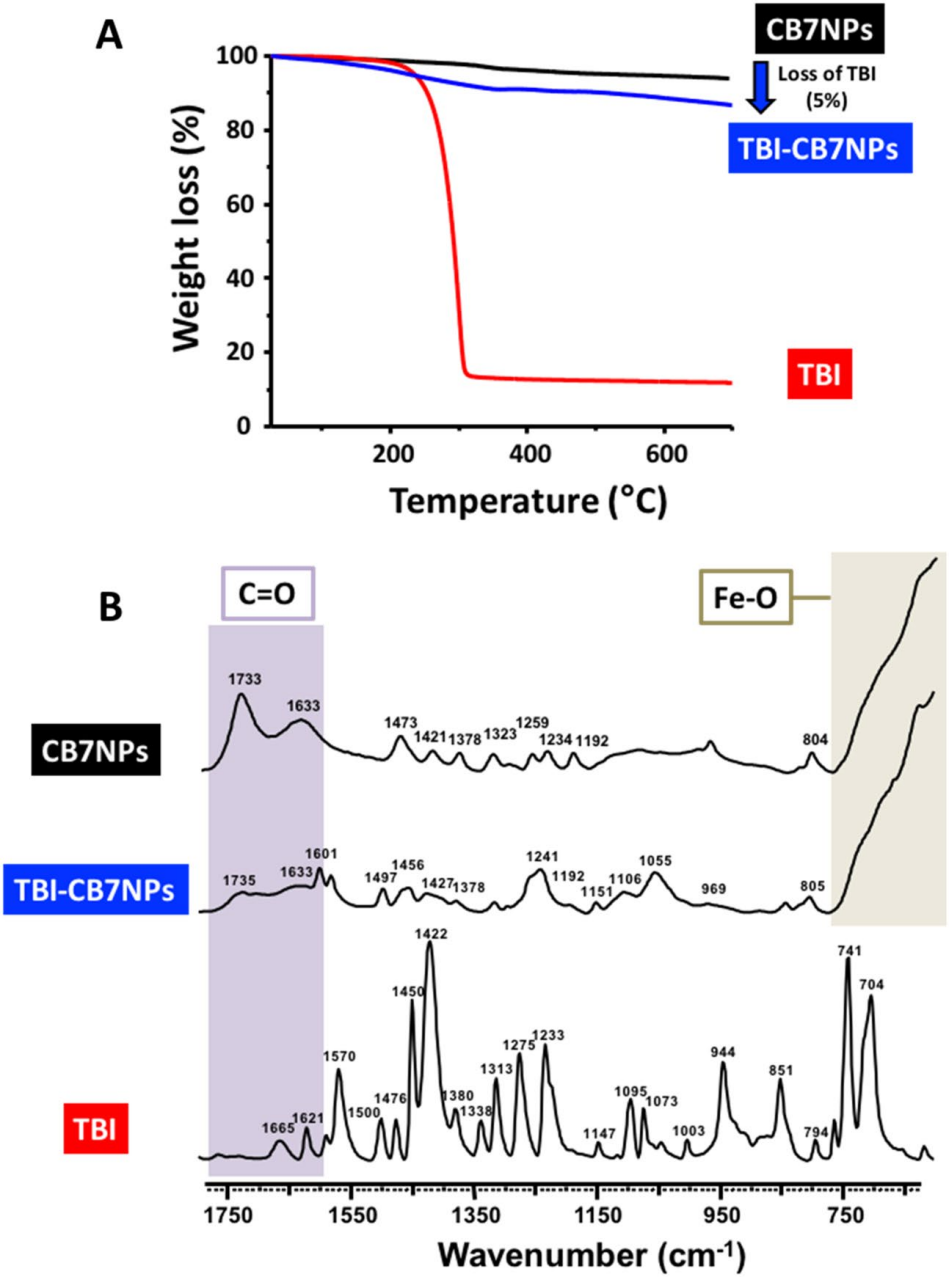
Thermo-gravimetric analysis (**A**) and FTIR spectra (**B**) of CB7NPs, **TBI**/CB7NPs, and **TBI**.

The fabrication of the nanocomposites is illustrated in Fig. 1. The γ-Fe_3_O_4_ nanomaterials were activated by conjugation to a host–guest complex of CB7 and **TBI** using similar approaches to those previously reported^17^. We mixed spherical CB7NPs (8 ± 1 nm in diameter) and **TBI** (CB7: **TBI**, 1:100) in water at pH 2 and room temperature for 24 h. The brown precipitate formed was collected with a magnet, washed several times with water to remove residual **TBI**, and analyzed by TGA and FTIR spectroscopy (Fig. 5).

The weight percentage of **TBI** interacting with CB7 on the surface of CB7NPs was determined by TGA. Figure 5a presents the weight losses of CB7NPs and **TBI**/CB7NPs. The more significant percentage loss occurs when **TBI**/CB7NPs are heated due to the loss of **TBI**. These data are consistent with the successful loading of CB7NPs with **TBI**. The TGA analysis of **TBI**/CB7NPs shows a composition of 90.95% iron oxide, 4.96% CB7, and 0.69% **TBI** corresponding to a 1:1 guest: host interaction (i.e., a 1:1 **TBI**: CB7 interaction, see Fig. S2 and Tables S2 and S3 in the Supporting Information for more details). The FTIR spectrum of **TBI**/CB7NPs is not simply the sum of its parts, which strongly suggests the presence of interactions between **TBI** and CB7NPs^17^. The spectrum of **TBI**/CB7NPs displays various peaks that can be attributed to ν(C–C) and ν(C–H) vibrations of CB7 as well as ν(C=C) and ν(C–H) vibrations of **TBI**. In addition, the FTIR spectrum of **TBI**/CB7NPs reveals a broadening of the peaks at 1733 and 1633 cm^−1^ corresponding to ν(C=O) vibration of the carbonyl portal of CB7. This observation suggests the presence of **TBI** in the CB7 cavity.

**Figure 6:**
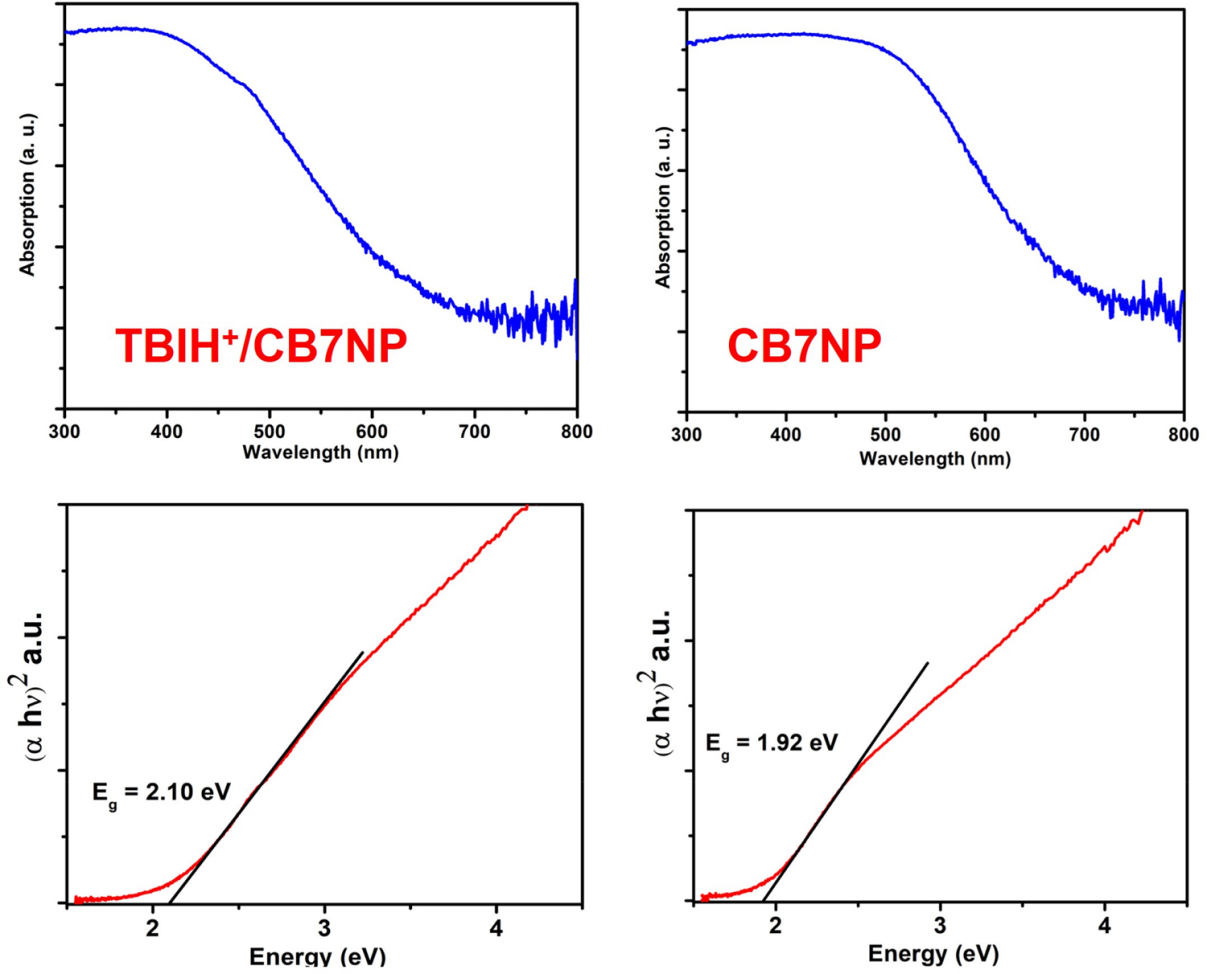
Diffusive-reflectance spectra (**DRS**) of CB7NPs and **TBIH**^**+**^/CB7NPs, and their Tauc plots suggest direct optical bandgaps.

The changes in the solid UV spectra (e.g., a shift in the band gap from 1.9 to 2.1 eV) also confirm the loading of **TBI** on CB7NPs (when pH was also changed from 7 to 2), Fig. 6. The Tauc plots clearly show different band gap values between the **TBIH**^**+**^/CB7NPs (E_g_ = 2.10 eV) and only CB7NPs (E_g_ = 1.92 eV).

**Figure 7:**
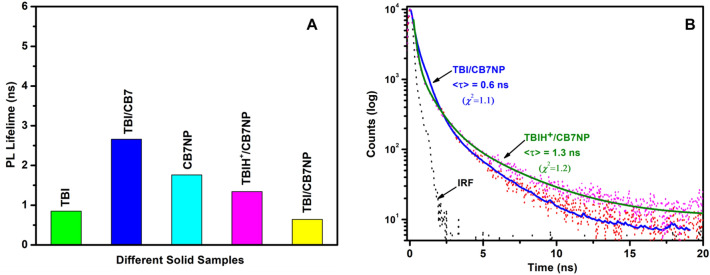
Excited-state lifetime average values as calculated using the equation in the Method section (**A**) and excited-sate lifetime plots (**B**) of different solid samples: **TBIH**^**+**^/CB7NPs, **TBI**/CB7NPs, CB7NPs, **TBI**/CB7, and **TBI**. Lifetime measurements were performed at 298 K, *λ*_ex_ = 375 nm, and *λ*_obs_ = 480 nm. IRF is the instrument response function shown in grey color.

As control experiments, the **TBI**/CB7 solid complex was prepared by grinding method (see “Methods” section). PL and TRPL were measured for all solid samples **TBI** (500 nm and 0.85 ns) and **TBI**/CB7 (452 nm and 2.7 ns), as shown in Fig. 7 and Fig. S3 in the Supporting Information. PL and excited-state lifetime measurements of solid samples for **TBI** upon inclusion inside CB7NPs (425 nm and 1.7 ns) were also measured at pH 2 (420 nm and 1.3 ns) and pH 7 (452 nm and 0.6 ns), as shown in Figs. 7 and S4 in the Supporting Information. Clear, distinct values are observed, which confirms the solid interactions. The blue-shift of ~ 50 nm and the increase in the lifetime value for **TBI**/CB7 when compared to the emission and lifetime decay from the free **TBI** (from 0.85 to 2.7 ns; Fig. S3) highlight the hydrophobic effects of the nonpolar cavity of CB7 in parallel to the results obtained in solution^25^. Similarly, an increase in the lifetime value (from 0.6 to 1.3 ns; Fig. S4) and a shift of ~ 25 nm was observed upon the protonation of **TBI** inside CBNPs (e.g., **TBIH**^**+**^/CB7NPs versus **TBI**/CB7NPs) because of similar polarity effects imposed by the more stable host–guest complex at lower pH values.

Noticeably, CB7NP (without TBI) has no pH-dependent emission spectra (Fig. S5 in the Supporting Information). Overall, the more accurate lifetime measurements demonstrated that the host–guest complex of CB7 renders the solid nanomaterial iron oxide more switchable through “non-covalent” interaction.

**Figure 8:**
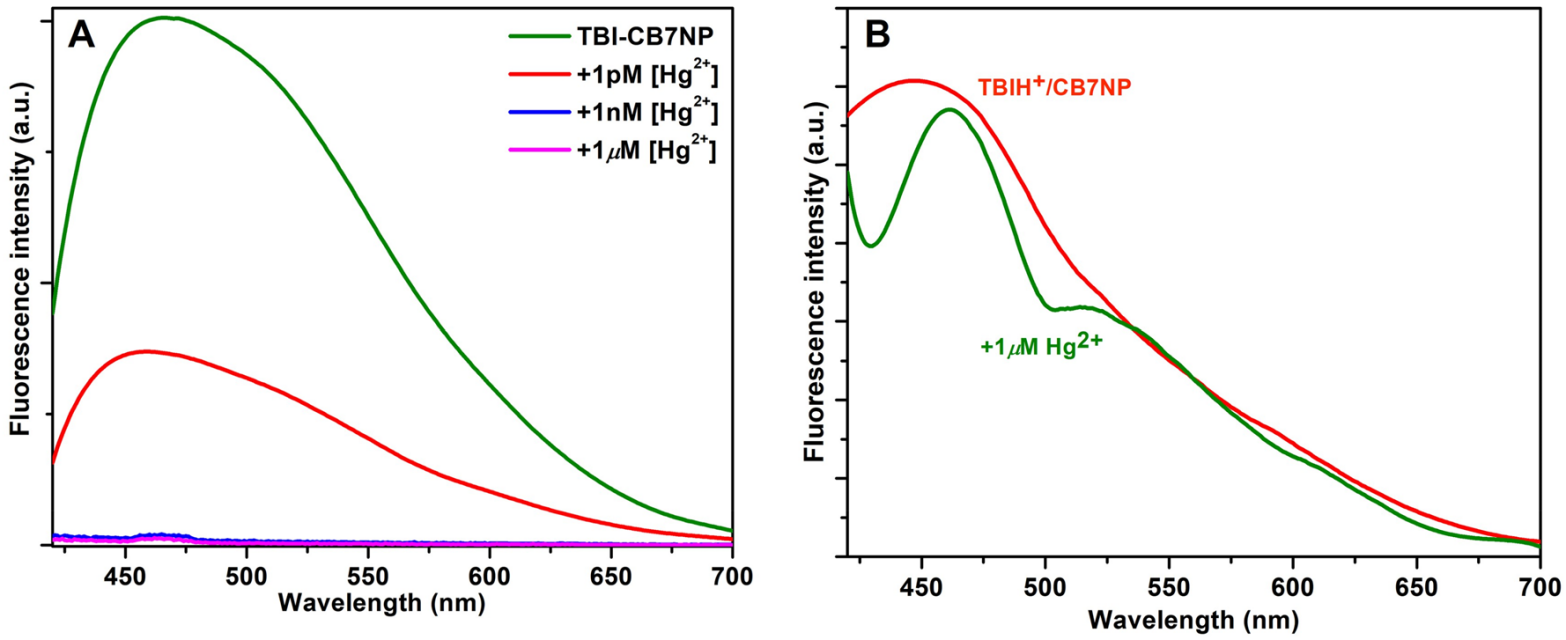
Fluorescence spectra of **TBI**/CB7NP (**A**) and **TBIH**^**+**^/CB7NP (**B**) with Hg^2+^ at different concentrations (as indicated directly in the graphs) and 298 K.

Thiophene was selected based on the expected soft–soft interactions between the sulfur atom and Hg^2+^^23^. The iron oxide magnetic nanoparticles were selected in particular to ease the collection of the magnetic materials by a magnet^17^. We, therefore, performed several experimental measurements to capture mercuric ions in the solid state. For example, we demonstrated in Fig. 8 that the interaction of **TBI**/CB7NPs with mercuric ions has caused a decrease in fluorescence intensity. The binding, in principle, can be regenerated upon changing the pH and using a magnet.

Moreover, the decay-associated spectra (DAS) analysis (Supporting Information, Fig. S6) revealed three excited-state lifetime components confirming the complex formation between **TBI**/CB7NP and mercuric ions in the solid state by analogy to the results in solution and the absence of nanoparticles^25^. In solution or solid-state, adding mercuric ions decreased the fluorescence intensity of **TBI** or **TBI**/CB7 in the water^25^ or on top of NP while keeping the excited-state lifetime unchanged (Table S4 in the Supporting Information). This advanced kinetic analysis unfolded different electronic states of **TBI**/CB7NPs and that only the shortest-living species (whose emission appears around 450 nm) was affected by mercury binding compared to others.

The repeated use of **TBI**/CB7NP for capturing mercuric ions is established by controlling the pH of the media, as manifested in the change in the fluorescence intensity. At neutral pH, the fluorescence intensity of **TBI**/CB7NP decreases when it binds a low concentration of Hg^2+^ in the solid state. However, **TBIH**^**+**^**/**CB7NP does not bind mercuric ions at acidic pH, and the fluorescence intensity is restored. The Hg^2+^-bound composite is easily separable with a magnet.

The long-term aim of the present research is to develop materials that are easy to handle, built, portable, and more importantly, to prove the concept of utilizing **TBI**/CB7 as a motif to significantly improve the turnover of the solid nanomaterials back to their initial state in response to pH. Figure 1 summarizes the flaws in the supramolecular approach employed by the **TBI**/CB7-functionalized γ-Fe_3_O_4_ NPs. At neutral pH, the ligands are primarily neutral and bind much less CB7 when compared to the protonated/cationic form (see the binding constants measured in Fig. 2). This explains the increase in the lifetime value of the CB7-modified NPs at low pH because of polarity effects. A subsequent increase in the pH of the tested water samples (that contain mercuric ions) substantially brings the ligand back to its neutral form. Our designed resetting mechanism is novel and has yet to be realized in literature, and it can be expanded to other ligands and analytes. The selected ligands are non-toxic, rendering the device disposable, as well.

## Conclusion

We modified magnetic NPs to capture mercuric ions from water samples at neutral pH. Specifically, the time-resolved optical behaviors of the CB7-modified NPs reveal a significant improvement in the turnover of their structures in response to pH because of the different binding affinities of CB7NP towards the protonated and neutral coordinating **TBI** ligand. Additionally, neutral and not protonated nanocomposites bind mercuric ions in water samples, enforcing the resetting mechanism. The most hazardous pollutant, mercury, in water resources is becoming of significant concern in many places where water contamination is critical in the quest for long-term economic and social stability.

Overall, the current approach is unique regarding the controllable separation of mercury ions using an external magnet and in response to pH through preferential binding of the host to guest molecules on the top of magnetic surfaces.

## Methods

### Samples

CB7 (purity > 99.9%) was produced by Sigma-Aldrich and used without any further purification. As instructed by Sigma-Aldrich, the calculated concentrations have considered the presence of 20% water in the supplied CB7 vials. The description for preparation and characterization of the **TBI**^23^ in the present study is included in the Supporting Information. Millipore water was used.

### Synthesis of CB7NPs

An aqueous solution (1 mL) of CB7 (n = 3 × 10^−5^ mol) was added to a colloidal suspension of NPs (4 mL, nFe = 7 × 10^−4^ mol) and transferred to a 10 mL microwave vessel with a crimp cap. The solution was heated by microwave irradiation of 2.45 GHz in a microwave reactor (CEM Discovery, CEM Inc. USA). The power was modulated to reach a temperature of 50 °C in 1 min and to maintain that temperature for 30 min. The maximum power applied was 300 W. Stirring was initiated at 50 °C during the heating cycle. Two heating cycles were used to prepare CB7NPs. The NPs were washed with water and precipitated by using a magnet. Iron concentration was deduced from UV–visible absorption data.

### **TBI**/CB7 complex synthesis

**TBI**/CB7 complex in the solid state was prepared by a grinding method, in which an equal amount of the two components were mixed and grounded for 20 min with acetone.

### **TBI**/CB7NPs preparation and characterizations

CB7NPs (nCB7 = 3 × 10^−4^ mol) and **TBI** (3 × 10^−3^ mol) were mixed in water (2 mL) and stirred for twenty-four hours at room temperature and pH = 7 to form inclusion complexes on the surface of NPs. The product was precipitated using a magnet and washed several times with water to afford **TBI**/CB7NPs. The presence of **TBI** was confirmed using FTIR spectroscopy. **TBI**/CB7NPs suspension (prepared above) was placed in water (2 mL) and stirred for 1 h at room temperature and pH = 2. The product was precipitated using a magnet and washed several times with water (pH 2) to afford **TBIH**^**+**^/CB7NPs.

### Hg^2+^-**TBI**/CB7NP preparation

**TBI/**CB7NPs (nCB7 = 3 × 10^−4^ mol) and Hg(OAc)_2_ salt (nHg^2+^  = 1 × 10^−3^ mol) were mixed in water (2 mL) and stirred for 1 h at room temperature and pH = 7 to form metal complexes on the surface of NPs. The product was precipitated using a magnet and washed several times with water to afford Hg^2+^*-TBI*/CB7NPs.

### Thermogravimetric analysis (TGA)

Solid samples (10 mg) under N_2_(g) flux were characterized with a SDT Q600 TA Instruments analyzer at a heating rate of 5 °C/min over a temperature range of 35–700 °C.

### Spectroscopy

UV–Visible absorption spectra were measured on a Cary-300 instrument (Varian). To estimate the binding constant, the total guest’s concentrations must remain unchanged while changing the concentration of the host molecules. The total concentration of the host is then plotted against the absorption or fluorescence intensity at a given wavelength. A Varian 400 MHz spectrometer measured NMR spectra in D_2_O in ppm against TMS reference. The addition of carefully chosen amounts of HCl (DCl) or NaOH (NaOD) controls the pH values of the solutions (± 0.2 units) as recorded using a pH meter (WTW 330i equipped with a WTW SenTix Mic glass electrode). The absorption spectra of the solid samples were obtained by using the Kubelka–Munk conversion (K–M = (1 − R)^2^/2R) of the recorded diffusive-reflectance spectra at room temperature for the solid samples on an FS5 spectrometer (Edinburgh, UK) equipped with an SC-30 (integrating sphere) as the sample holder. The specular reflection of the sample surface light was removed from the signal by directing the incident light at the sample at an angle of 0°; only the diffusive reflected light was measured. Polytetrafluoroethylene (PTFE) polymer was used as the reference. The bandgap energy (Eg) values of the solid samples from the DRS spectra were calculated using E_g_ = 1240 eV nm l^−1^, where l is the absorption edge (in nm). The solid-state photoluminescence (PL) measurements were carried out for the suspension samples after they were vacuum-dried over the demountable corvettes under a fume hood for twenty-four hours. The time-resolve photoluminescence (TRPL) spectra were collected using time-correlated single-photon counting (TCSPC) on a LifeSpec II spectrometer (Edinburgh Instruments) by using EPL-375 picosecond diode laser (*λ*_*e*x_ = 375 nm, repetition rate = 5 MHz, and instrument function = 30 ps) for excitation in the solid state. The monitored emission maxima were at 480 nm. The time-resolved emission (intensity of ~ 1000–3000 counts/s) was collected (up to 10,000 counts/s) by a red-sensitive high-speed PMT (Hamamatsu, H5773-04) detector. Marquardt–Levenberg algorithm opted to analyze the collected data utilizing the iterative reconvolution method to minimize *χ*^2^. The contribution of each lifetime, *τ*_i_ with an amplitude *α*_*i*_ in the multiexponential model, to the steady-state intensity was adjusted using the formula

$${f}_{i}=\frac{{\alpha }_{i}{\tau }_{i}}{\sum_{j}{\alpha }_{j}{\tau }_{j}} ,$$

where the sum in the denominator is over all the decay times and amplitudes. The average excited-state lifetime is then calculated by

$$\overline{\tau }=\sum_{i}{f}_{i}{\tau }_{i}$$

For the decay-associated spectra (DAS) measurements, emission decays collected every 10 nm over the entire emission spectra of the solid samples with a dwell time of 10 s at each wavelength were globally fitted to a tri-exponential model function and then convoluted with an instrument response function (IRF) of ~ 30 ps. The time-resolved data were specifically analyzed using the Edinburgh FAST software.

### Consent to participate

The participants consented.

## Supplementary Information

Supplementary Information.

## Data Availability

All relevant data are within the paper.
