# Supplementary material for: Separation of mercuric ions using 2-thienylbenzimidazole/cucurbit[7]uril/iron-oxide nanoparticles by pH control
Source: Sci Rep. 2023 Jul 12;13:11287. doi: 10.1038/s41598-023-38199-2 (PMC10338676; doi:10.1038/s41598-023-38199-2)
Supplement: Supplementary file 1 — Supplementary Information. [file 41598_2023_38199_MOESM1_ESM.docx]

**SUPPORTING INFORMATION**

Separation of Mercuric Ions using 2-Thienylbenzimidazole/Cucurbit[7]uril/Iron-Oxide Nanoparticles by pH Control

Falguni Chandra,^†§^ Paltan Laha, ^†§^ Farah Benyettou,^‡^  Tina Skorjanc,**^‡^** and Na’il Saleh*^†§^

^†^Department of Chemistry, College of Science, United Arab Emirates University, P.O. Box 15551, Al Ain, United Arab Emirates

^§^Zayed Bin Sultan Center for Health Sciences, United Arab Emirates University, PO. Box 15551 Al Ain, United Arab Emirates

^‡^New York University Abu Dhabi, P.O. Box 129188, Abu Dhabi, United Arab Emirates

**Supporting methods section**

**
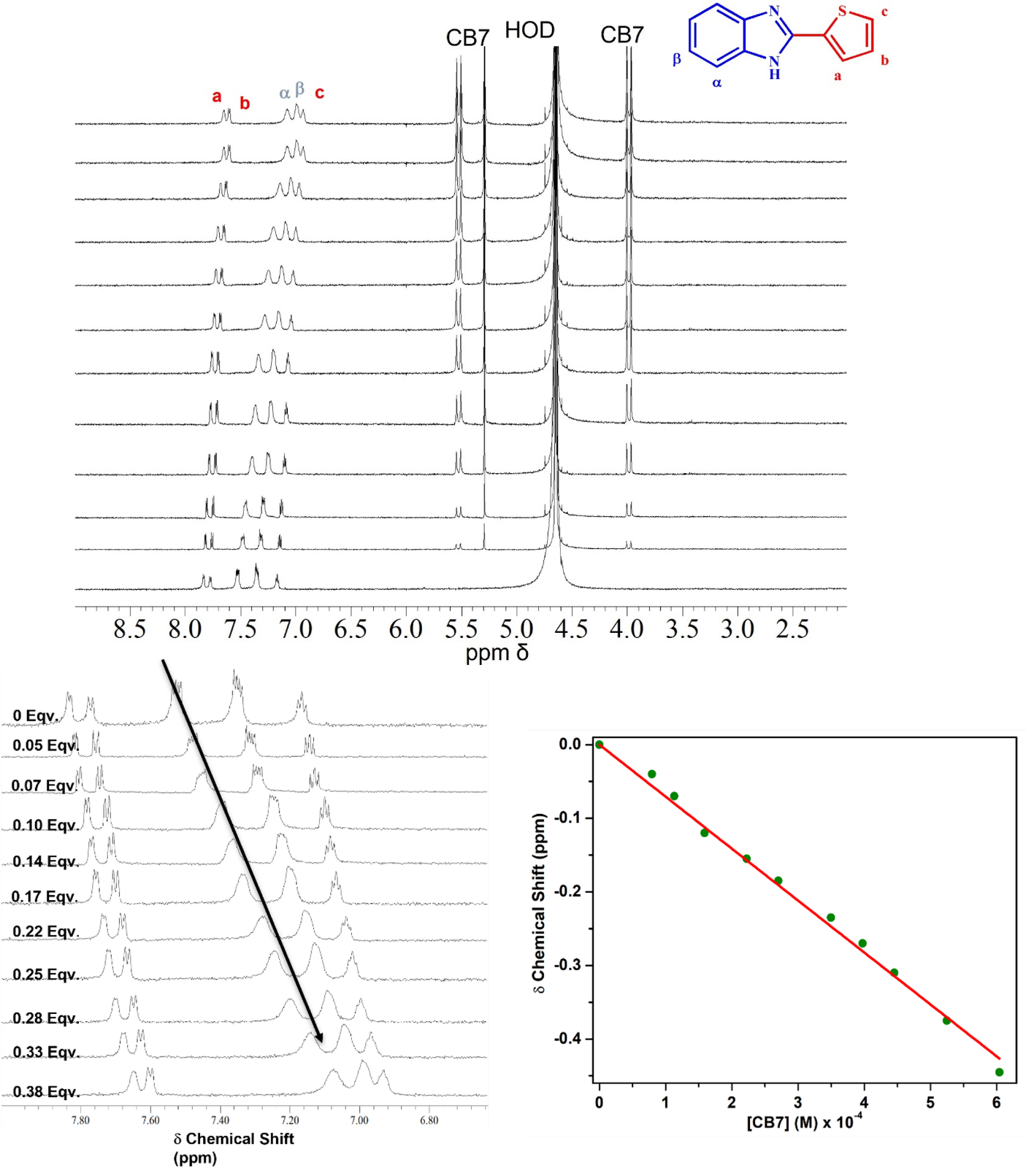
**

**Figure S1.** ^1^H NMR titration of **TBI** (1.6 mM) in D_2_O (400 MHz) with CB7 at pD 2.0 (0–1.0 equivalent). Due to the poor solubility, measurements at pD 7 were not possible. The inset shows the nonlinear fitting plot of chemical shift (ppm) from 7.1 to 7.5 *versus* the concentration of CB7 in molarity. *K* was evaluated as (1.1± 0.001) × 10^4^ M^−1^

**Table S1.** Binding constant data and the CB7-induced p*K*_a_ shifts of **TBI** in aqueous solutions.

| **System (method)** | **p*K*_a_** | **p*K’*_a_**  **_(with CB7)_** | **∆p*K*_a_** | ***K* _TBI/CB7NP_**  **(10^6^ L/mol)** | ***K* _TBIH_^+^_/CB7NP_**  **(10^6^ L/mol)** |
| --- | --- | --- | --- | --- | --- |
| **TBI** and CB7 (PL) | 4.6^[17]^ | 5.5^[17]^ | 0.9^[17]^ | 0.015^[17]^ | 0.052^[17]^ |
| **TBI** and CB7 (UV) | 4.4 | 7.7 | 3.3 | 0.243 | 2.85 [480]^a^ |
| **TBI** and CB7 (NMR) | - | - | - | - | 0.011 |

^a^ Calculated from the thermodynamic relation in Figure 1.


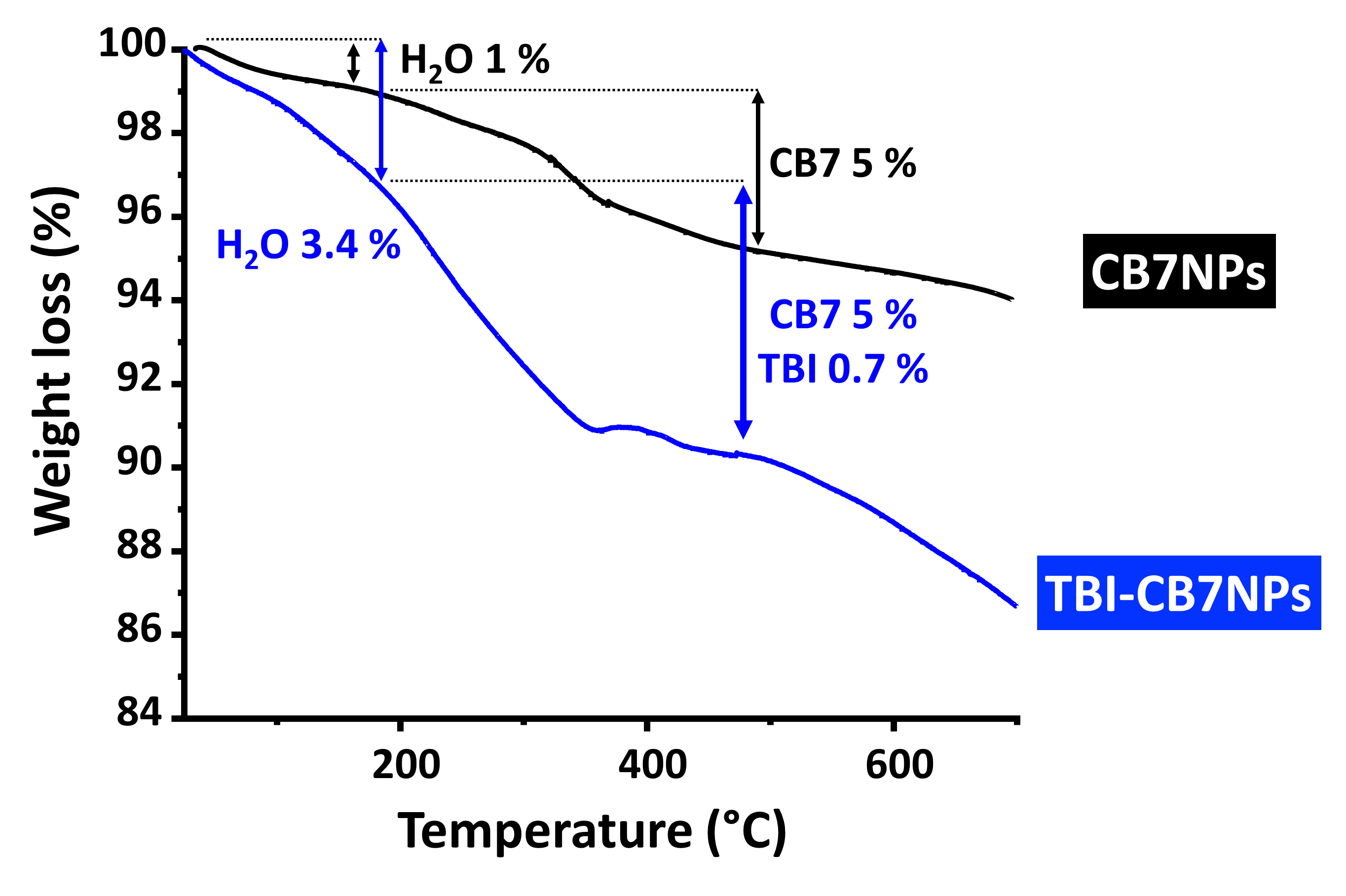


**Figure S2.** Thermo-gravimetric analysis of CB7NPs (black) and **TBI**/CB7NPs (blue), showing the weight losses of **TBI**, CB7NPs, and **TBI**/CB7NPs.

**TGA Calculations**

TGA percentages were used to estimate the weight % of **TBI** and CB7 in the powder. We divided the weight % by the molecular weight, giving us several moles (Tables S2 and S3). We then compared the number of moles of **TBI** with CB7, which allowed us to conclude to 1:1 **TBI**: CB7 interaction on the nanoparticle’s surface.

**Table S2.** TGA calculations for CB7NPs.

|  | Weight loss (%) | MW (g.mol^-1^) | n (mol) |
| --- | --- | --- | --- |
| **H_2_O** | 1.1 | 18.015 | 0.061 |
| **NPs** | 94 | 159.69 | 0.591 |
| **CB7** | 4.9 | 1162.96 | 0.004 |

**Table S3.** TGA calculations for **TBI**/CB7NPs.

|  | Weight loss (%) | MW (g.mol^-1^) | n (mol) |
| --- | --- | --- | --- |
| **H_2_O** | 3.4 | 18.015 | 0.188732 |
| **NPs** | 90.95 | 159.69 | 0.572 |
| **CB7** | 4.96 | 1162.96 | 0.004 |
| **TBI** | 0.69 | 194.23 | 0.004 |

**
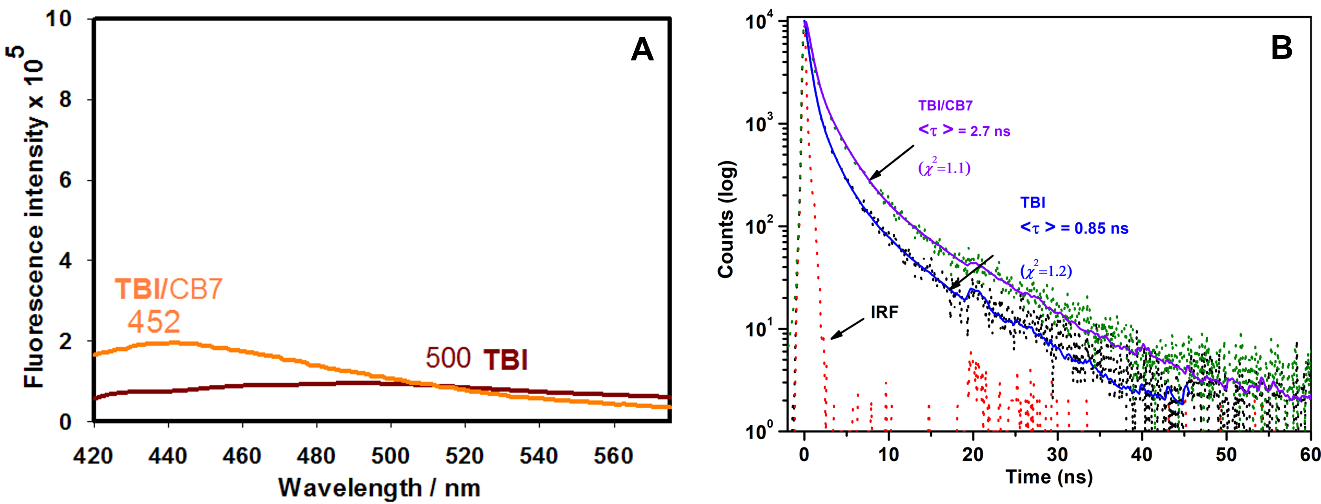
**

**Figure S3.** (A) Fluorescence solid state spectra (SSF) and (B) lifetime emission traces of different solids: **TBI** free and upon inclusion inside CB7. Lifetime measurements were performed at 298 K, *λ*_ex_ = 375 nm, and *λ*_obs_ = 480 nm. The average excited-state lifetime values (see Methods section) are indicated directly in the graph.

**
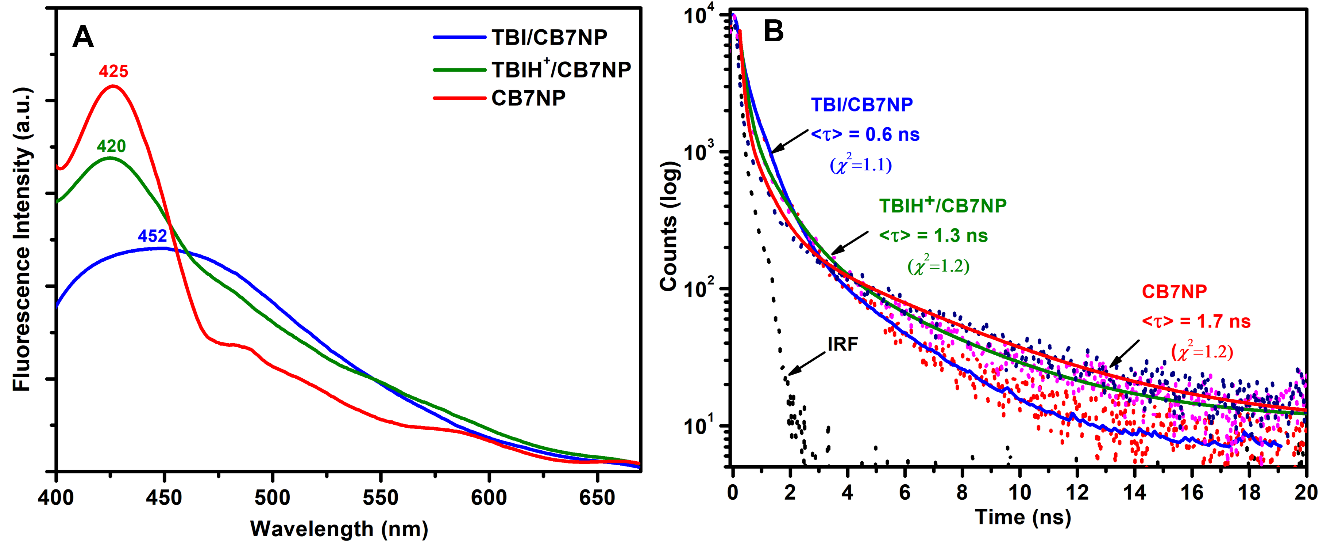
**

**Figure S4.** (A) Fluorescence solid state spectra (SSF) and (B) Lifetime emission traces of different nanocomposites: **TBIH^+^**/CB7NPs, **TBI**/CB7NPs, and CB7NPs.

Lifetime measurements were performed at 298 K, *λ*_ex_ = 375 nm, and *λ*_obs_ = 480 nm. The average excited-state lifetime values (see Methods section) are indicated directly in the graph.


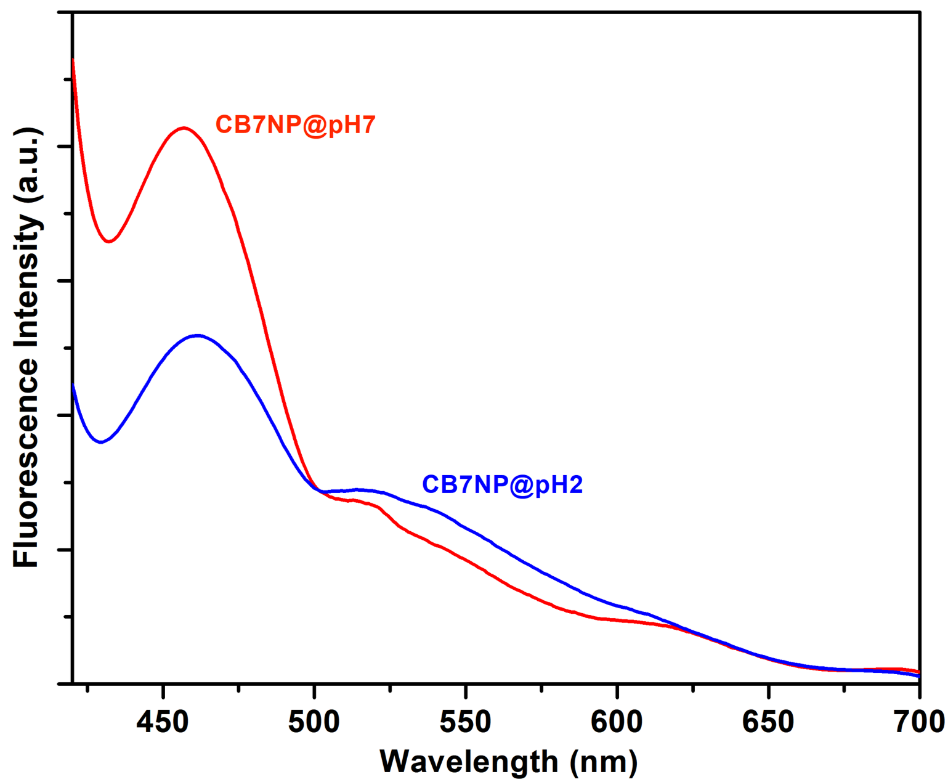


**Figure S5:** Fluorescence spectra of CB7NP at two different pH


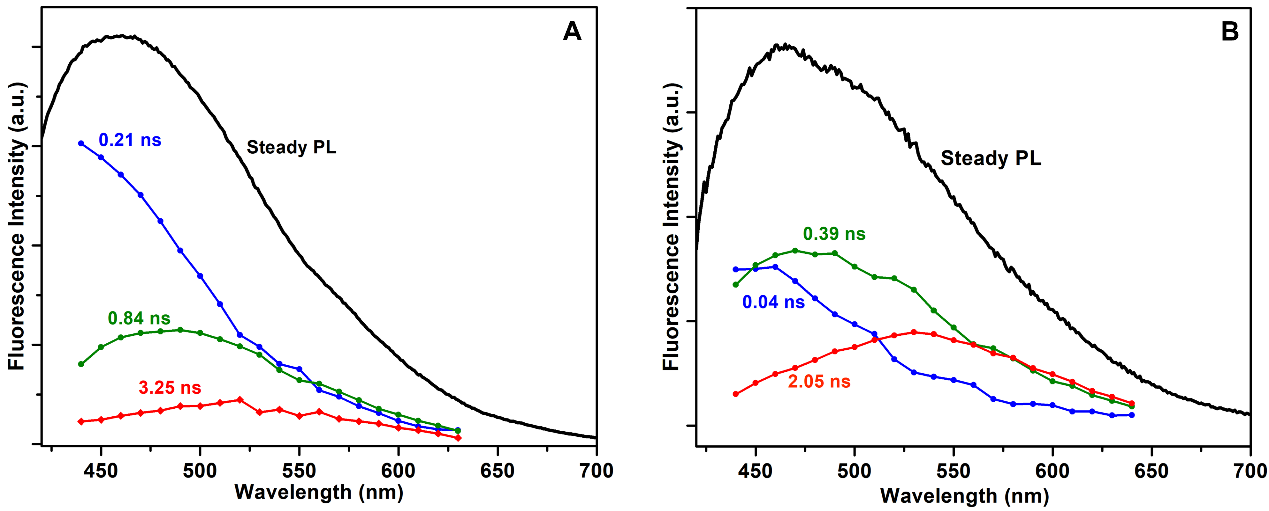


**Figure S6.** Decay-associated spectra (DAS) of a three-component mixture of fluorophores for (A) **TBI**/CB7NP and (B) 1*u*M Hg^2+^ + **TBI**/CB7NP in the solid state upon excitation at 375 nm and at 298 K. The corresponding steady-state spectra of each solid are also shown for comparison (Experimental Section).

**Table S4.** Excited-state lifetime results of different NPs performed at 298 K, *λ*_ex_ = 375 nm, and *λ*_obs_ = 480 nm.

| **Complex** | ***τ* −Individual (ns)** | **Contribution %** | ***<τ>***  **(ns)** | ***χ^2^*** |
| --- | --- | --- | --- | --- |
| **TBI**/CB7NP | 0.14  0.60  2.6 | 46.03  40.78  13.17 | 0.65 | 1.1 |
| 1pM Hg^2+^+**TBI**/CB7NP | 0.15  0.55  2.39 | 48.22  38.70  13.08 | 0.59 | 1.2 |
| 1nM Hg^2+^+**TBI**/CB7NP | 0.04  0.44  2.06 | 34.76  47.14  18.10 | 0.59 | 1.0 |
| 1μM Hg^2+^+**TBI**/CB7NP | 0.11  0.49  2.18 | 41.04  44.96  14.00 | 0.57 | 1.1 |

**Preparation of 2-(thiophen-2-yl)-1H-benzimidazole (TBI) ligand**

A solution of *O*-phenylenediamine (0.01 mol) in absolute ethanol (5 mL) was added in small portions to a solution of thiophene-2-carbaldehyde (0.01 mol) in absolute ethanol (5 mL). The reaction mixture was irradiated in CEM Discovery microwave reactor (Irradiation - 2.45 GHz). The power applied was 300 W for 7 minutes at 50^0^C. TLC monitored the reaction. The precipitate was collected by filtration, washed with water, dried, and recrystallized from a methanol/hexane solvent system (yield: 83%). ^1^H NMR (400 MHz, DMSO-d6): δ 12.91 (s, 1H), 7.80 (d, *J* = 4.0 Hz, 1H), 7.69 (d, *J* = 4.0 Hz, 1H), 7.52 (d, *J* = 35.2 Hz, 2H), 7.21 – 7.19 (m, 1H), 7.16 (s, 2H). ^13^C-NMR (100 MHz, DMSO-*d_6_*): δ 111.5-147.5 (Ar-C)

**Figure S7.** The ^1^H NMR spectra of **TBI** in DMSO-*d_6_*.

**
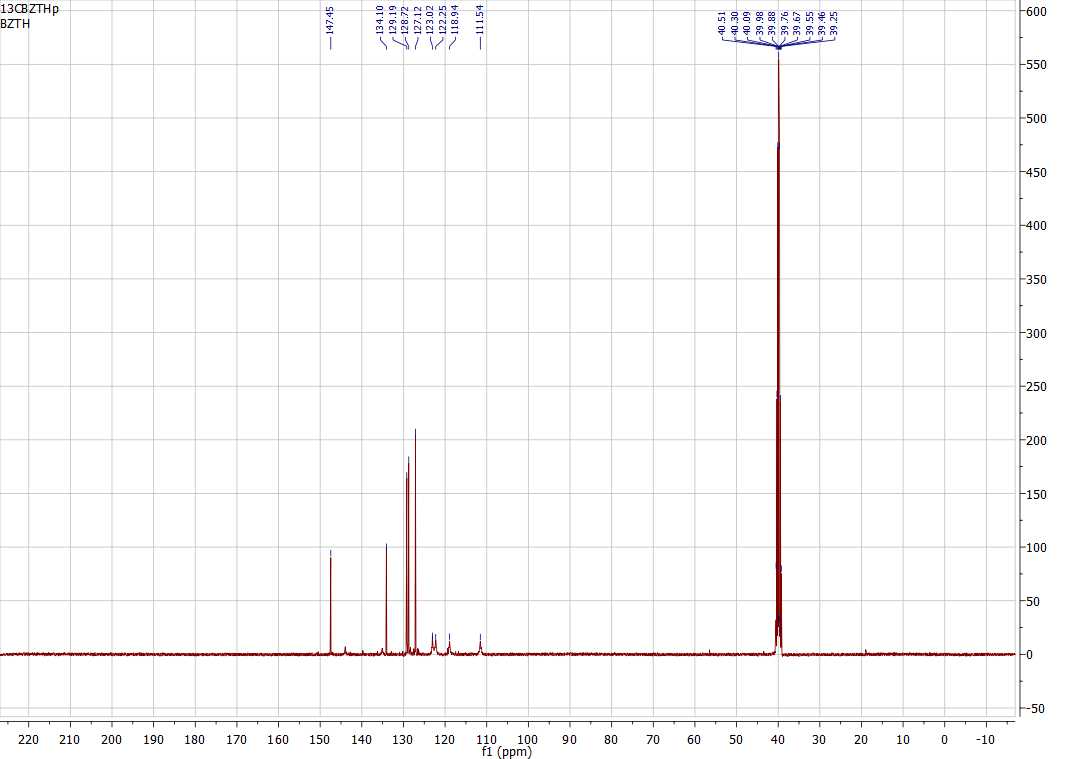
**

**Figure S8**. The ^13^C NMR spectra of **TBI** in DMSO-*d_6_*.
